# PK2/PKR1 Signaling Regulates Bladder Function and Sensation in Rats with Cyclophosphamide-Induced Cystitis

**DOI:** 10.1155/2015/289519

**Published:** 2015-12-22

**Authors:** Biao Chen, Huiping Zhang, Lili Liu, Jiaojiao Wang, Zhangqun Ye

**Affiliations:** ^1^Department of Urology, Tongji Hospital, Tongji Medical College, Huazhong University of Science and Technology, Wuhan 430030, China; ^2^Institute of Urology, Tongji Hospital, Tongji Medical College, Huazhong University of Science and Technology, Wuhan 430030, China; ^3^Family Planning Research Institute, Tongji Medical College, Huazhong University of Science and Technology, Wuhan 430030, China; ^4^Department of Pathology, Wuhan Women and Children's Medical Center, Wuhan 430016, China

## Abstract

Prokineticin 2 (PK2) is a novel chemokine-like peptide with multiple proinflammatory and nociception-related activities. This study aimed to explore the potential role of PK2 in modulating bladder activity and sensation in rats with cyclophosphamide- (CYP-) induced cystitis. Changes of PK2 and prokineticin receptors (PKRs) in normal and inflamed urinary bladders were determined at several time points (4 h, 48 h, and 8 d) after CYP treatment. Combining a nonselective antagonist of prokineticin receptors (PKRA), we further evaluated the regulatory role of PK2 in modulating bladder function and visceral pain sensation via conscious cystometry and pain behavioral scoring. PK2 and prokineticin receptor 1 (PKR1), but not prokineticin receptor 2, were detected in normal and upregulated in CYP-treated rat bladders at several levels. Immunohistochemistry staining localized PKR1 primarily in the urothelium. Blocking PKRs with PKRA showed no effect on micturition reflex activity and bladder sensation in control rats while it increased the voiding volume, prolonged voiding interval, and ameliorated visceral hyperalgesia in rats suffering from CYP-induced cystitis. In conclusion, PK2/PKR1 signaling pathway contributes to the modulation of inflammation-mediated voiding dysfunction and spontaneous visceral pain. Local blockade of PKRs may represent a novel and promising therapeutic strategy for the clinical management of inflammation-related bladder diseases.

## 1. Introduction

Interstitial cystitis/painful bladder syndrome (IC/PBS) is a chronic pathological condition of the bladder characterized by symptoms such as pelvic pain, urgency or frequency in urination, and suprapubic discomfort [1]. IC/PBS inevitably influences normal physical and mental health and presents a remarkable negative effect on the quality of life of patients [1]. People with IC/PBS constantly feel discomfort at normal bladder pressure, suggesting abnormal excitability of their micturition reflex pathway [2, 3].

Etiologically, several hypotheses, including epithelial dysfunction, latent infection, neurogenic inflammation, and autoimmune phenomena, have been proposed; however, the exact pathogenesis of IC/PBS remains largely unclear [1–3]. Recently, the regulatory role of bioactive molecules associated with inflammation and pain sensation in the emergence of IC/PBS has received increasing research attention [3–6]. Histological investigations have demonstrated some degree of inflammatory invasion in the majority of bladder biopsies from IC/PBS patients [3, 7]. These studies strongly support the idea that inflammation-relevant factors are implicated in bladder dysfunction and visceral hypersensitivity during IC/PBS.

Prokineticin 1 and prokineticin 2 (PK1 and PK2) are the mammalian orthologs of Bv8 (amphibian peptide* B. variegata* 8) and mamba intestinal toxin 1 (MIT1), which were isolated from skin secretions of* Bombina variegata* and mamba venom, respectively [8]. PK1 and PK2 represent a novel chemokine-like family characterized by the conserved N-terminal sequence “AVITGA” and a distinctive motif consisting of 10 cysteine residues [9, 10]. Two G-protein-coupled receptors, prokineticin receptor 1 (PKR1) and prokineticin receptor 2 (PKR2), are responsible for delivering signals carried by PK1 and PK2 into effector cells [11].

In the past decade, the biological activities of these peptides have been the subject of extensive research, implying that PK2, but not PK1, participates in the physiological or pathological processes of inflammation and nociception [8]. Earlier observations have confirmed the overexpression of PK2 in multiple activated immune cells, inflamed tissues, and organs showing multiple proinflammatory activities via PKR1 [12–15]. For example, an investigation by Martucci et al. shows that PK2 stimulates macrophages to secrete inflammatory cytokines while reducing anti-inflammatory cytokine production [15]. In addition, the pivotal role of PK2/PKR1 interaction in nociception and inflammation-related hyperalgesia has also been determined, in which the transient receptor potential vanilloid receptor 1 (TRPV1) serves as a downstream responsive element [16–18].

To date, numerous biologically active substances have been demonstrated to modulate bladder function and micturition reflex. Early observations have implicated cytokines, neuropeptides, and growth factors in the regulation of inflammation-related bladder dysfunction [2–5]. Although previous investigations have revealed the presence of PK2 transcripts in urinary bladder tissues [9], the physiological role and expression profile of PK2 cognate receptors in the bladder remain unknown. Considering the proinflammatory activity and nociception facilitation property of PK2, we attempted to elucidate the expression of PK2 and prokineticin receptors (PKRs) in the urinary bladder during CYP-induced cystitis in rats. Moreover, by combining a nonselective PKR antagonist, we explored the potential role of PK2 in modulating voiding function via conscious cystometry (CCM) and visceral pain scoring.

## 2. Materials and Methods

### 2.1. Animals

Female Sprague-Dawley rats (220–270 g) were purchased from the Experimental Animal Center, Huazhong University of Science and Technology, Wuhan, China. The rats were housed with free access to food and water under standard laboratory conditions. The Institutional Animal Care and Use Committee of Tongji Medical College approved all the experiments and animal use. All the experimental procedures were performed in accordance with the National Institutes of Health* Guide for the Care and Use of Laboratory Animals*.

### 2.2. Induction of Cystitis

Intraperitoneal (i.p.) injection of CYP (Sigma-Aldrich) was applied to establish a cystitis animal model. Control rats received saline injections. Single-dose CYP (150 mg/kg i.p.) injection was performed to induce acute bladder inflammation (4 h or 48 h duration), and a low-dose CYP (75 mg/kg) was injected i.p. every 3 days to mimic chronic bladder inflammation until the eighth day. The rats used for CCM studies received CYP (150 mg/kg i.p.) treatment for 48 h.

### 2.3. Section Preparation and Immunohistochemistry (IHC)

After deparaffinization and rehydration, 5 *μ*m cross sections were heated in boiling ethylene diamine tetraacetic acid buffer (0.01 M, pH 8.0) under high pressure for 2 min to retrieve antigens. Following blockade with skim milk, sections were incubated in PKR1 antibody solution (1 : 800, BS2957, Bioworld Technology) at 4°C overnight. A diaminobenzidine substrate kit (GK500705, Gene Technology) was used to detect the target proteins. Sections incubated in the absence of primary antibody and in the primary antibody preabsorbed with PKR1 peptide (5 *μ*g/mL) were processed to evaluate antibody specificity.

### 2.4. RNA Preparation and Real-Time RT-PCR

Tissue mRNA was extracted with Trizol reagent (15596-026, Invitrogen). For each sample, 3 *μ*g of RNA was reverse transcribed into cDNA. Real-time RT-PCR was then performed in a 20 *μ*L reaction system with the following thermal profiles: initial denaturation at 95°C for 2 min followed by 40 cycles of denaturation at 95°C for 15 s and extension at 58°C for 30 s. All amplification procedures were performed in triplicate to ensure reliability of results. Melting curve analysis and electrophoresis were performed to ensure the specificity of the primers. Primer sequences obtained from previous studies are shown in Table 1 [14, 16, 17]. *β*-Actin served as internal control, and the relative expression levels of* PK2* and* PKR1* mRNA in CYP-treated samples were calculated using the 2^−ΔΔCt^ method.

### 2.5. Enzyme-Linked Immunosorbent Assay (ELISA)

Frozen bladder samples were homogenized (1 : 2, weight/volume) in 0.01 M phosphate-buffered saline (pH 7.4) solution supplemented with 10 *μ*L/mL protease inhibitor cocktail (P8340, Sigma) on ice. Lysates were centrifuged twice at 10,000 ×g for 30 min at 4°C. Total protein concentrations were quantified with a protein assay kit (23227, Thermo Fisher Scientific). PK2 was quantified using a rat PK2-specific ELISA kit (E-EL-R0780c, Elabscience) in accordance with the manufacturer's instructions. PK2 amount of each sample was calculated from linear standard curves and converted to the content in total protein. Supernatants were not diluted, and no samples fell below the detection limit of the kit.

### 2.6. Evaluation of Visceral Pain Behavior

Exceptional care was taken by the researchers to be silent during pain behavior observations, and the rats were allowed to adapt to the atmosphere before testing based on our previous experience [19]. The rats underwent visceral pain behavior evaluation for 2 h before CCM. We quantified the nociceptive behavior with a modified pain behavioral scoring scale as follows: normal = 0, piloerection = 1, labored breathing = 2, ptosis = 3, licking of the abdomen = 4, and limp body/rounded back = 5. Each behavior lasting over a 1 min period was recorded and summed for an overall pain score (maximum score = 15). Following intravesical instillation of PKRA (1 *μ*M, Zhou's laboratory) or vehicle (1% dimethylsulfoxide, DMSO, in saline), the same rats were evaluated after CCM. PKRA is a small-molecule PKR antagonist synthesized in Zhou's laboratory [20, 21]. In the present study, PKRA was compounded as previously reported with some modifications [20, 22]. PKRA yields IC_50_ values of approximately 12 nM for PKR1 and 18 nM for PKR2, as measured by Ca^2+^ luminescent assay in CHO cells. Additionally, PKRA is insoluble in water and freely soluble in DMSO.

### 2.7. CMM

Intravesical catheter implantation was performed in 24 rats as previously described [23]. For CCM, rats were maintained in a plastic dorsal access restrainer, which was placed over multilayer filter papers to collect the voiding liquid. The preimplanted catheter was then connected to a T-junction to the vesical pressure channel of urodynamics system (UDS-600, Laborie Medical Technologies). The left port of the T-connector was linked to a syringe pump that consistently instilled the bladder with saline to elicit micturition. On the eighth day following catheter implantation, all the rats received CCM to establish basal data. Subsequently, half of them were subjected to CYP treatment (150 mg/kg i.p.), with the remaining rats as controls. To determine the potential role of PK2 in modulating bladder activity under physiological and pathological conditions, CMM was performed again in the control rats (*n* = 12) after 15 min of intravesical perfusion with approximately 200 *μ*L of vehicle (*n* = 6) or PKRA (1 *μ*M) solution (*n* = 6), whereas CYP-treated rats (*n* = 12) received CCM before and after intravesical irrigation of vehicle (*n* = 6) or PKRA (1 *μ*M) solution (*n* = 6) for 30 min. A minimum of six micturition cycles were recorded for each rat. The instillation rate was set at 10 mL/h for CCM. Once voiding commenced, we immediately recorded the time points to calculate the voiding interval (VI). Filter papers were weighed using an electronic balance. The weight of the voiding liquid was converted into voiding volume (VV) with a scale of 1 g = 1 mL. The concentration of PRKA used in the current study was empirically determined according to pilot studies. In these pilot studies, we estimated several concentrations (200 nM, 1 *μ*M, and 5 *μ*M). PRKA concentration of 200 nM showed no effect, whereas the highest concentration (5 *μ*M) exhibited similar effects to that of 1 *μ*M. Thus, 1 *μ*M PKRA was selected for the present study.

### 2.8. Statistics

Data are expressed as mean ± SE for all values in this study. Statistical significance among groups was detected by ANOVA or repeated-measures ANOVA (CCM data), followed by Bonferroni post hoc tests. Significance was considered at *P* < 0.05.

## 3. Results

### 3.1. Real-Time RT-PCR

**Figure 1:**
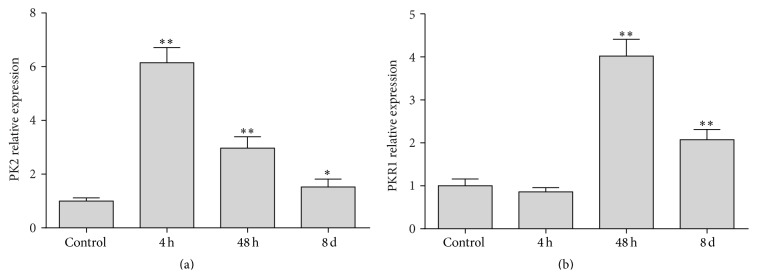
Time-dependent changes of PK2 and PKR1 expression in whole urinary bladders following CYP treatment with varying duration. (a)* PK2* mRNA levels were significantly increased at all time points after CYP administration, compared with the controls. (b)* PKR1* mRNA levels at 48 h and 8 d time points increased significantly, compared with those of the controls and 4 h time points. *∗∗* indicates *P* < 0.01; *∗* indicates *P* < 0.05. *n* = 6-7 for each group.

CYP treatment significantly elevated the* PK2* mRNA levels of all inflamed bladders (Figure 1(a)). Compared with those of the controls (*n* = 7), the* PK2* mRNA levels in 4 h rats (*n* = 6, *P* < 0.01), 48 h rats (*n* = 6, *P* < 0.01), and 8 d rats (*n* = 6, *P* < 0.05) were increased 6.15-, 2.97-, and 1.53-fold, respectively, after CYP injection. Compared with those of the controls (*n* = 7), the* PKR1* mRNA levels were significantly increased 4.02-fold (*P* < 0.01) and 2.07-fold (*P* < 0.01) in bladders with 48 h (*n* = 6) and 8 d (*n* = 6) CYP-induced cystitis, respectively, but not in the bladders with 4 h cystitis (*n* = 6) (Figure 1(b)). However,* PKR2* mRNA was not detected in all samples.

### 3.2. IHC

**Figure 2:**
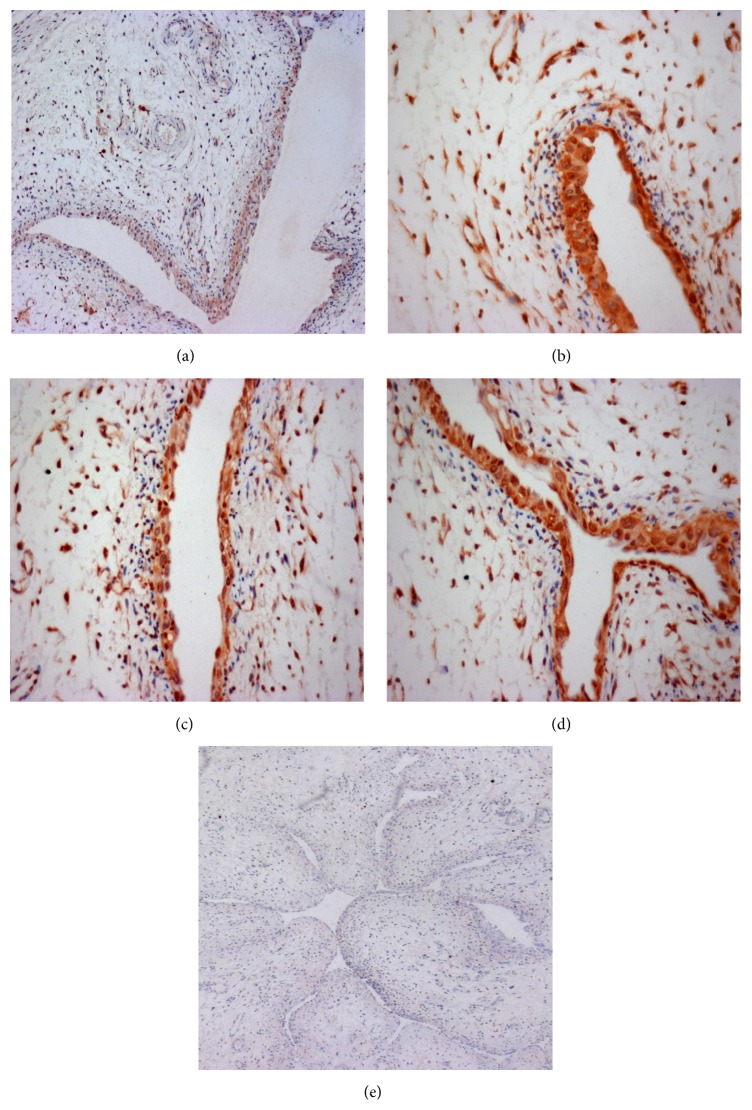
PKR1 immunohistochemical staining in the bladder sections ((a) control; (b) 4 h; (c) 48 h; (d) 8 d). PKR1 protein expression was primarily detected in the urothelium and markedly increased in all rats that suffered from CYP-induced cystitis regardless of duration. (e) A section serves as the negative control incubated with the primary PKR1 antibody absorbed onto the blocking peptide. Sample size *n* = 5–7 for each group. Magnification: (a, b, c, d) 10x; (e) 4x.

PKR1 was predominantly expressed in the urothelium and dispersively distributed in the lamina propria in all rats (Figures 2(a)–2(d)). CYP treatment markedly upregulated the PKR1 expression in the urothelium of the bladders with 4 h, 48 h, and 8 d cystitis (Figures 2(b), 2(c), and 2(d)). The detrusor smooth muscle exhibited no PKR1 immunoreactivity in all bladders. Increased infiltration of mononuclear cells was also observed in all inflamed bladders. Incubation with blocking peptide by the preabsorbed primary antibody reduced the PKR1 immunoreactivity to controlled levels (Figure 2(e)). When the primary antibody was absent, no positive immunostaining was observed (data not shown).

### 3.3. ELISA

**Figure 3:**
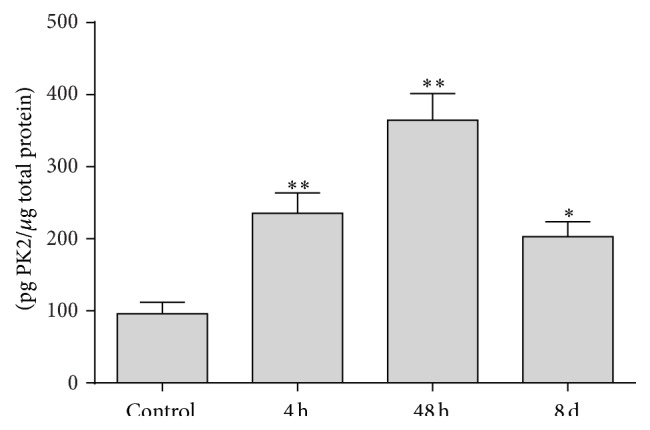
ELISA experiments showed that the PK2 protein expression was increased in all bladders following CYP treatment. The PK2 protein levels increased significantly at each time point after CYP injection relative to those of the control bladders (no CYP). Sample size *n* = 5–7 for each group. *∗∗* indicates *P* < 0.01; *∗* indicates *P* < 0.05.

The PK2 protein level (pg/*μ*g total protein) in the bladders with 48 h cystitis (*n* = 6) was determined to be the most robust (364.6 ± 36.87) and significantly increased (*P* < 0.01) one, compared with that of the control (95.93 ± 16.06). As summarized in Figure 3, PK2 was also significantly increased to 235.5 ± 28.2 and 202.8 ± 20.93 in the inflamed bladders with 4 h (*n* = 7, *P* < 0.01) and 8 d (*n* = 5, *P* < 0.05) CYP-induced cystitis, respectively.

### 3.4. Effect of PKRA on Spontaneous Visceral Pain

**Figure 4:**
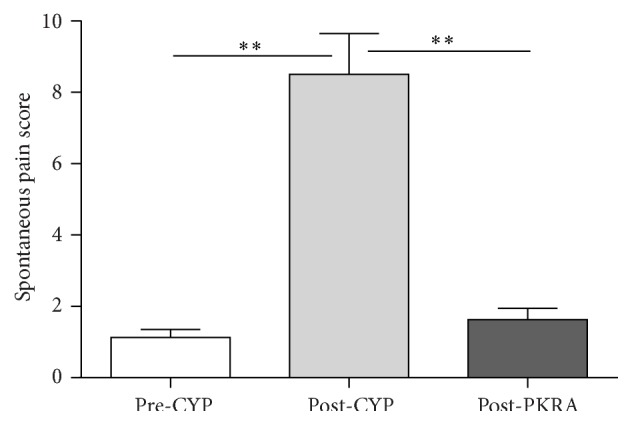
Evaluation of the effect of PKRA on reducing spontaneous visceral pain evoked by CYP treatment in rats. Intravesical administration of PKRA (1 *μ*M) significantly reduced spontaneous pain in rats with 48 h duration of CYP-induced cystitis. *∗∗* indicates *P* < 0.01. Sample size *n* = 6 for each group.

CYP treatment significantly (*P* < 0.01) increased the spontaneous visceral pain behavior in all rats (*n* = 12) with 48 h cystitis, indicating an increase in the mean visceral pain score from 1.13 ± 0.23 (before CYP) to 8.50 ± 1.15 (after CYP) (Figure 4). Intravesical perfusion of PKRA (1 *μ*M) significantly (*P* < 0.01) reduced the visceral pain sensation and reversed the pain behavior score to 1.63 ± 0.32 in the same rats (*n* = 6, Figure 4). Nevertheless, no significant change in nociceptive behavior was observed in the control rats (*n* = 6) that received vehicle perfusion (data not shown).

### 3.5. Effect of PKRA on Bladder Function

#### 3.5.1. Control Rats (No CYP Treatment)

**Figure 5:**
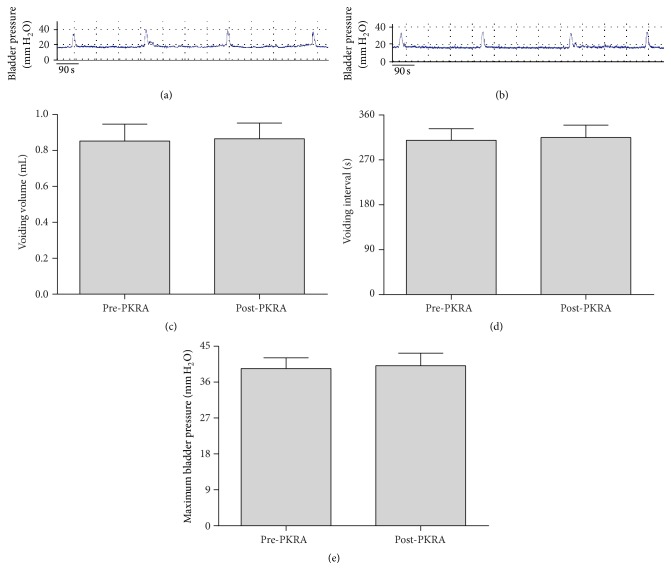
Effect of PKRA on modulating bladder function examined by cystometry in conscious normal rats (no CYP). (a) and (b) are original recordings of pressure-volume curve in a control rat before PKRA (a) and after PKRA (b) treatment, respectively. PKRs blocking with PKRA showed no effect on urodynamic parameters examined including voiding volume (c), voiding interval (d), and maximum bladder pressure (e) in control. Sample size *n* = 6 for each group.

To investigate whether intravesical application of PKRA physiologically modulates bladder function, we performed CCM on the control rats before and after vehicle (*n* = 6) or PKRA treatment (*n* = 6) (Figures 5(a) and 5(b)). Drug administration with PKRA (1 *μ*M) exerted no effect on the urodynamic parameters, including VV, VI, and maximum bladder pressure (MBP) in rats without cystitis (Figures 5(c), 5(d), and 5(e)). Similar results were observed in the rats that received vehicle irrigation (data not shown).

#### 3.5.2. Cystitis Rats (48 h)

**Figure 6:**
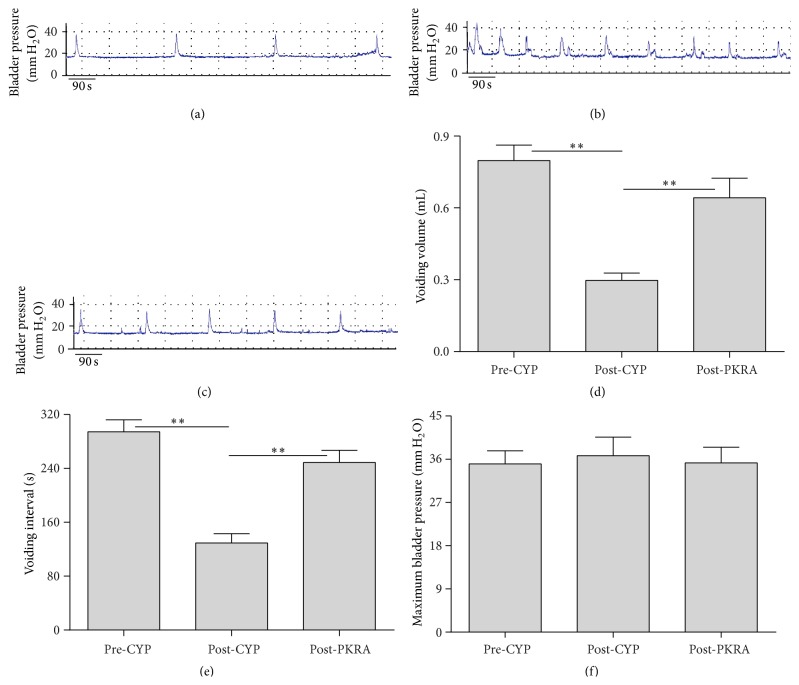
Effect of PKRA on bladder function in rats with 48 h CYP-induced cystitis. (a), (b), and (c) represent the pressure-volume curves for the same rat at different time points after CYP administration: (a) pre-CYP; (b) post-CYP; (c) post-PKRA. Summary showed intravesical infusion of PKRA (1 *μ*M) significantly increased voiding volume (d) and voiding interval (e) with no effect on maximum bladder pressure (f). *∗∗* indicates *P* < 0.01. *n* = 6 for each group.

CCM was performed on all 12 rats prior to CYP injection to establish baseline data (Figure 6(a)). CYP treatment generated instable pressure-volume curves (Figure 6(b)) and significantly (*P* < 0.01) decreased VV and VI from 0.80 ± 0.064 mL and 294.3 ± 17.96 s to 0.30 ± 0.031 mL and 129.3 ± 13.85 s, respectively (Figures 6(d) and 6(e)). Subsequent application of PKRA (1 *μ*M) stabilized the bladder (Figure 6(c)) in six rats and significantly (*P* < 0.01) recovered VV and VI to 0.64 ± 0.081 mL and 248.8 ± 18.14 s, respectively (Figures 6(d) and 6(e)). In addition, no significant change in MBP was observed in these rats at all time points (Figure 6(f)). Nevertheless, irrigation of bladder with vesicle failed to stabilize the inflamed bladder (*n* = 6, data not shown).

## 4. Discussion

In the present study, we demonstrate for the first time the expression of both PK2 and PKR1, as well as the location of PKR1 in the urinary bladder of rats with or without cystitis (Figures 1–3). As a response to CYP treatment, the expression of both PK2 and PKR1 in the inflamed bladders significantly increased at the genetic and protein levels (Figures 1–3). In the context of cystitis, the inhibition of PKRs with PKRA not only alleviated inflammation-related visceral pain (Figure 4) but also significantly improved bladder dysfunction (Figure 6). However, topical administration of PKRA at the bladder level exerts no effect on normal micturition in the control rats, indicating the inaction of the PK2/PKR1 signal pathway on the urinary bladder under physiological conditions (Figure 5). Overall, our study provides new insights into the mechanisms underlying the occurrence of hypersensitivity and detrusor overactivity during inflammation-associated bladder disorders. Moreover, PK2/PKR1 signaling may represent a novel target to treat inflammation-related diseases of the urinary bladder.

In mammalians, PK2 is present in various organs where it underlies diverse biological activities [20, 24]. Recently, several studies reported about the expression of PK2 and PKRs in various epithelial tissues [25, 26]. We also detected the basal expression of PKR1 in the urothelium of the controls; however, blockage of PKRs with PKRA exhibited no effect on the activity of micturition reflex in these rats. This result suggests that PKR1-mediated signal does not contribute to the modulation of the bladder function and sensation under normal conditions. The absence of an effect may be ascribed to relatively low concentrations of PK2 in the urothelium under normal conditions, indicating that the level of PK2 is not sufficiently high to activate cognate receptors and consequently transmit biological signals. Further studies focusing on the distribution of PK2 in the urinary bladder can help elucidate the regulatory role of PK2 in micturition reflex under physiological conditions.

After i.p. injection, CYP is metabolized to acrolein, which directly comes into contact with the urothelium and causes severe cystitis accompanied with inflammatory cell infiltration [19]. Several published investigations have demonstrated the existence of PK2 in various leukocytes, as well as its overexpression in inflammation sites [12–15, 27]. Thus, the infiltrated and activated immune cells may be hypothesized to at least partly contribute to the increase in PK2 in the bladder after CYP treatment. In addition, the urothelium itself or other components of the bladder can potentially produce and secrete PK2 when stimulated by noxious substances such as acrolein.* In vitro*, PK2 promotes the migration, survival, and differentiation of monocytes; it also facilitates the production of proinflammatory cytokines IL-1 and IL-12 while reducing the release of anti-inflammatory chemokines in a PKR1-dependent manner in monocytes [12, 13, 15].* In vivo*, systematic administration of Bv8 significantly increases the peripheral leukocyte counts and the IL-1*β* level of splenocytes in mice [12, 28]. These findings suggest that PK2/PKR1 signaling promotes, at least in part, the occurrence and progression of inflammation in the bladder after CYP administration.

The expression of PKR1 in the urothelium is upregulated after CYP treatment, as indicated by our IHC data, as a response to inflammatory stimulation (Figure 2). Similar results were reported in studies on other tissues, including the epithelium, under physiological and/or pathological conditions [25, 29–31]. CYP administration can cause immune cells to infiltrate into the bladder wall, suggesting that the immigrating and activated immune cells possibly contribute to the overexpression of PKR1 in cystitis as PKR1 is expressed on these cells. However, the detailed mechanisms need to be further elucidated. Nevertheless, the increase of PKR1 expression in the urothelium following CYP administration enhanced the modulatory effect of PK2/PKR1 signaling on bladder function and sensation during cystitis.

The role of the PK2/PKR1 signaling pathway in modulating inflammatory response, including inflammation-mediated visceral pain, has been widely studied [13, 16–18, 32]. The precise mechanisms may not be completely known, but several studies support the notion that locally elevated PK2 reduces the nociceptive sensation threshold by stimulating the release of proinflammatory cytokines from activated immune cells, in addition to the production of downregulated anti-inflammatory factors [15, 28, 33]. PK2 is also likely to modulate pain perception by directly activating PKRs on nociceptive sensory neurons involving a functional interaction with the colocated TRPV1, the key effector of various noxious stimuli [13, 16–18, 34]. In addition, the expression of TRPV1 on the urothelial cell surface, as well as its important role in bladder sensation and regulation during cystitis, has been well demonstrated previously [35]. In the current study, elevated PK2 may have directly simulated the nerve plexus system underlying the urothelium and increased the sensory signal input via the afferent limb, causing visceral hyperalgesia and bladder hyperactivity. PK2 may have also diffused to the bladder lumen and activated PKR1 on the urothelial cells, thereby modulating the bladder sensory pathway. These speculations are consistent with our findings that intravesical application of PKRA can improve inflammation-related bladder overactivity and cystitis-mediated pain behavior in rats.

Further studies are still needed as PKR2 is also expressed in the dorsal root ganglion neurons. The development of a more selective inhibitor and a specific* PKR2* null animal model can help accurately define the signal pathway by which PK2 regulates inflammation-related bladder hypersensitivity and overactivity. Additionally, the precise mechanisms by which PK2 signaling regulates bladder sensation and functions under inflammatory conditions remain unexplored. Nonetheless, our study provides new insights into the modulation of bladder function and sensation during cystitis. Improved understanding of the alternations in the bladder during cystitis increases the possibility of developing enhanced methods for the treatment.

## 5. Conclusion

Both PK2 and PKR1 are physiologically expressed in the rat urinary bladder, and CYP-induced cystitis significantly upregulates their expression levels. Functional studies show that PK2/PKR1 signaling is involved in the regulation of bladder hypersensitivity and visceral hyperalgesia during cystitis. Pharmacological inhibition of PKRs shows a potential clinical value for the treatment of inflammation-associated bladder disorders such as IC/PBS.

## Figures and Tables

**Table 1:** Real time qPCR primer sequences.

Gene	Type	Sequence
Pk2	Forward	CAAGGACTCTCAGTGTGGA
	Reverse	AAAATGGAACTTTCCGAGTC

Pkr1	Forward	CGCACCGTCTCCCTCTAC
	Reverse	GTTTGACACTTCATCCGCG

Pkr2	Forward	TGTCCACCCCTTAAAACGGA
	Reverse	TCAAGCCCGAAGACGAAGAG

*β*-actin	Reverse	CGTTGACATCCGTAAAGACCTC
	Reverse	TAGGAGCCAGGGCAGTAATCT
